# The impact of sport on the physical, psychological and social wellbeing of people with chronic breathlessness: A mixed-methods systematic review

**DOI:** 10.1177/02692155231190770

**Published:** 2023-07-30

**Authors:** Callum Bradford, Denis Martin, Kirsti J Loughran, Noelle Robertson, Alexandra Carne, Nathan Skidmore, Samantha L Harrison

**Affiliations:** 1 School of Health and Life Sciences, 5462 Teesside University, Middlesbrough, UK; 2 School of Psychology and Vision Sciences, University of Leicester, Leicester, UK

**Keywords:** Chronic obstructive pulmonary disease, sport, systematic review, chronic breathlessness, mixed-methods

## Abstract

**Objective:**

Sport participation may have quality-of-life benefits for people with chronic breathlessness; however, its feasibility and impact on health are unknown. We aimed to synthesise the scientific literature concerning the impact of sport for people with chronic breathlessness.

**Data sources:**

Searches of MEDLINE, CINAHL, PsycINFO, Embase, SPORTDiscus and Google Scholar were conducted (May 2023).

**Review methods:**

Studies were included if they assessed the impact of sport with participants who were likely to suffer from chronic breathlessness due to an underlying condition (e.g. severe asthma, heart failure). A convergent-segregated approach to synthesis in accordance with the JBI methodology for mixed-methods reviews was utilised, including meta-analytic and meta-aggregation analyses.

**Results:**

A total of 22 studies met the inclusion criteria. Studies sampled 1017 participants from 13 different countries, with sample sizes ranging from 5 to 185. Causes of breathlessness consisted of chronic respiratory diseases (9 studies) and coronary heart disease (13 studies). Design-wise, 18 reported quantitative methods, 3 qualitative, and 1 mixed-methods.

**Conclusions:**

Sports were well-adhered to with only minor/unrelated adverse events reported. Improvements in exercise capacity were observed although there was no impact on health-related quality of life. Other quantitative outcomes extracted varied widely across studies, making it difficult to draw firm conclusions. Participation in sports was reliably recorded at intensity consistent with moderate-to-vigorous activity despite being self-paced. Qualitative themes emphasised the positive elements of sport participation, namely, social cohesion, the capacity to incorporate culture, and the idea that participation is enjoyable rather than a necessary chore to maintain one’s health.

## Introduction

Chronic breathlessness refers to breathlessness that persists despite optimal treatment of the underlying pathophysiology.^1^ Approximately 85% of cases are accounted for by asthma, congestive heart failure, chronic obstructive pulmonary disease (COPD), psychogenic disorders, pulmonary fibrosis, and pneumonia.^2^ Around 9%–13% of people will experience chronic breathlessness,^3,4^ with incidence rising with age to 37% for those aged over 60 years.^5^

Cardiopulmonary rehabilitation, consisting of exercise and education, is recommended for everyone with breathlessness,^6^ and its benefits are unequivocal.^7–9^ However, there are five notable shortcomings evidenced across the published literature, namely, restricted access (<25% of potentially eligible patients are referred),^10^ poor engagement (30–50% do not attend initial assessment),^10,11^ low completion rates (≈30%),^12^ limited influence on behaviour change relating to physical activity,^13^ and poor sustainability of any benefits gained (most subside by 12 weeks).^14^ However, if patients with COPD complete their rehabilitation programmes and subsequently sustain exercise, the downward trajectory of lung capacity, health status and quality of life can be mitigated.^15^

Explanations for participant attrition include a perceived lack of benefit,^16–18^ living alone,^16–18^ travel distance,^17^ and psychological wellbeing.^19,20^ Sport participation in a team or group setting may have the potential to address some of these limitations: sports offer greater accessibility (with over 100,000 sports facilities in England comfortably exceeding around 200 pulmonary rehabilitation locations),^21,22^ they are more inherently enjoyable (compared to the gym which has been described as ‘very boring’ and ‘monotonous’),^23^ and their non-hospital context may be less likely to provoke memories of previous exacerbations and any attendant trauma cognitions.^24^ Sport has a profound ability to draw upon previous life experiences, with many often describing sport clubs as a fundamental part of their culture, community and identity.^25^ For older adults, participation in sport has been shown to improve psychological wellbeing,^26,27^ quality of life,^26^ and physical functioning.^28,29^

Unfortunately, those with breathlessness may have difficulty participating in most sports safely, comfortably or confidently given their condition. Constraints of this kind have created a growing interest in sports that are altered to cater for those with limited mobility and endurance to encourage long-term engagement in exercise, notably walking football,^23^ dance groups^30^ and Nordic walking.^31^ However, to date, the available evidence underpinning the benefits, adherence and safety of sports for people with chronic breathlessness has not been synthesised. Therefore, this review aims to identify and synthesise literature concerning the impact of organised sport on the physical, psychological and social wellbeing of people with chronic breathlessness.

## Methods

The Preferred Reporting Items for Systematic Reviews and Meta-Analyses (PRISMA) guidelines^32^ and the Template for Intervention Description and Replication (TIDieR) framework were used to structure the reporting of this review.^33^ The review has been conducted with a mixed-methods design, utilising a convergent-segregated approach to synthesis and integration in accordance with the JBI methodology for mixed-methods systematic reviews.^34^ This systematic review was registered at the International Prospective Register of Systematic Reviews (PROSPERO) with the protocol number CRD42021250679.

### Eligibility criteria

#### Population

Adults affected by chronic breathlessness^1^ or likely to suffer from chronic breathlessness as a direct result of an underlying condition (e.g. COPD, persistent and severe asthma, pulmonary fibrosis, heart failure). Covid-19 was not listed as one of these underlying conditions given its relationship with long-term breathlessness is not well understood.^35^ However, if a study involving Covid patients explicitly reported chronic breathlessness, then it would be considered eligible for inclusion.

#### Intervention

*Organised sports*. Sports were defined for the purpose of this review as organised activities that involve physical exertion, utilise sport-specific skills and rules, and have the *capacity* to be competitive. However, this capacity did not have to be applied for an intervention to be included. For example, football drills would be included, and a competitive match need not be played. Sports that are played in their most recognised, unmodified format and those that are modified to cater for less able populations were also included, i.e. both association and walking football. Sports with a meditative focus, i.e. meditative movement (e.g. tai chi, yoga), and ‘exer-gaming’ or e-sports interventions were excluded because reviews of meditative movement and video game–based rehabilitation for breathless populations have already been conducted elsewhere recently.^36,37^ Sports had to be performed in-person (not virtually, or home-based) and in a group setting. Sports comprised the main intervention, rather than being adjunct to or part of another intervention such as a training camp.

#### Comparisons

Comparisons include usual care, education or an active control such as pulmonary rehabilitation.

#### Outcomes

Studies were eligible if they included an outcome assessing the physical, psychological and/or social wellbeing of participants. Studies measuring the feasibility and intensity of sports for those with breathlessness were also included. Qualitative studies met the inclusion criteria if they explored the experiences and views of participants, wellbeing, potential motivations or triggers, factors that lead to sustainability and evaluations of the sport.

#### Study design

Both qualitative and quantitative designs were included. Specifically, randomised control trials, quasi-randomised controlled trials, one-group pre–post designs, mixed-methods, and qualitative studies were all within the inclusion criteria. Published abstracts were included where details of the full trial/study were not available. Non-intervention studies such as reviews and book chapters were excluded.

### Information sources and search strategy

Free-text keywords and index terms were combined with Boolean operators for comprehensive searches of MEDLINE (EBSCOhost), CINAHL (EBSCOhost), PsycINFO (EBSCOhost), Embase (OVID), SPORTDiscus (EBSCOhost), and Google Scholar. A grey literature search was also conducted via OpenGrey, as well as reference list and citation searches. The search strategy for MEDLINE is outlined in Appendix A and was adapted for the other databases. The search was initially conducted in August 2021, and updated in September 2022 and May 2023.

### Selection of studies and data extraction

A proportion of the studies elicited through the search (*n* = 1104; 16.9%) were screened for inclusion by CB and AC via titles and abstracts, discrepancies (<1%) were resolved by consensus between the authors, and the remaining articles (*n* = 5444) were screened by CB. A total of 48 full-text articles were later screened by CB and AC individually, with discrepancies resolved by consensus. The identification and screening of studies via other methods (e.g. grey literature, reference lists, citation searching) were conducted by CB.

Data extraction was conducted by one author (CB) and checked by another (NS). The TIDieR checklist^33^ was consulted to aid data extraction, including details surrounding the geographical location, design, sample, cause of breathlessness, sports intervention, outcomes measured, phenomena of interest, and descriptions of the main findings. For qualitative studies, themes and illustrations were extracted to assign levels of plausibility.

### Quality assessment

Methodological quality was assessed by two independent reviewers (CB and NS), with discrepancies resolved by discussion. Quantitative trials were assessed via a modified Downs and Black quality assessment checklist.^38^ For qualitative designs, a Critical Appraisals Skills Programme checklist^39^ was applied, consisting of 10 questions. Quantitative studies were given an overall rating of poor (0–10), fair (10–19), good (20–25) or excellent (26–28). Qualitative studies were not totalled as an overall rating, as recommended in the guidance.^39^

### Synthesis

A meta-analysis exploring the impact of sport for people with breathlessness was conducted if a consistent outcome was measured post-intervention in five or more controlled studies, applying a random-effects model.^40^ The restricted maximum likelihood estimator was used to calculate the heterogeneity variance $\tau^{2}$ .^41^ A Knapp–Hartung adjustment was used to calculate the confidence intervals around the pooled effects.^42^ Standardised mean differences were utilised so studies which measured similar outcomes with different scales could be pooled together. The reported standardised mean differences are for Hedges’ adjusted g. The remaining outcomes subsumed within the inclusion criteria but not meeting the conditions for meta-analysis are outlined in a narrative summary.

The qualitative data extracted were combined in a meta-aggregation.^43^ A three-step thematic analysis approach was applied. Step one involved the extraction of all findings from all included studies, each accompanied with a level of plausibility, judged on how accurately a finding is represented by its corresponding illustrations. Plausibility ratings range from unequivocal, credible or not supported. Plausibility ratings are judged subjectively via an evaluation of the illustrations and to what extent the overall finding encapsulates them. Step two involves the merging of findings into succinct categories, with each category comprising at least two findings. Step three similarly encompasses the creation of synthesised findings, each of which consist of two or more categories.^43^ Categories and synthesised findings were established by CB and SH.

The quantitative and qualitative syntheses were juxtaposed to create a third, overall configured analysis as outlined by the JBIs manual for mixed-methods systematic reviews.^34^ This juxtaposition of evidence led to the formation of lines of argument which could address the phenomenon of interest in a more holistic manner. This integration is interwoven throughout the discussion section.

## Results

**Figure 1.**
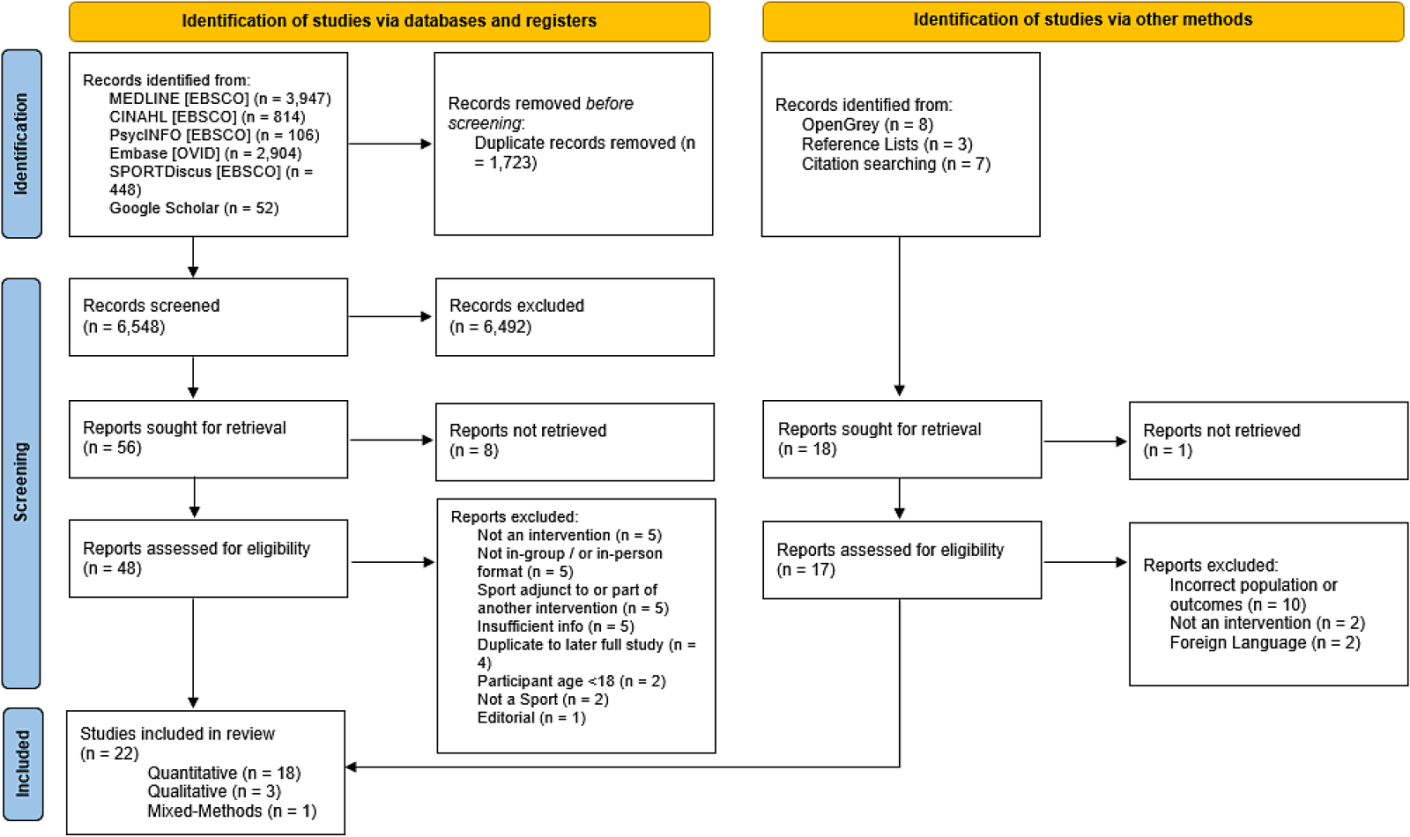
PRISMA flow diagram,^32^

The database searches yielded 8271 results, with a final total of 22 studies included after duplicates were removed, sifting, and additional sources (i.e. reference lists and citation searches) checked^30,44–64^ (see Figure 1). The summary of scores from the TIDieR checklist and characteristics of the included studies can be found in Tables 1 and 2, respectively. An outline of the extracted findings is described in Appendix B. Findings from the appraisal of studies are summarised in Tables 3 and 4.

**Table 1.** Summary of scores for TIDieR.

TIDieR items	
1. Description of the name of the intervention	100%
2. Description of the intervention rationale, theory or goal of the elements essential to the intervention	86%
3. Description of materials used in the intervention	100%
4. Detailed description of procedures used in the intervention	86%
5. Description of the person who provided the intervention	50%
6. Description of the modes of delivery	68%
7. Description of the location where the intervention occurred	45%
8. Description of the parameters regarding the intervention	95%
9. Was the intervention tailored, i.e. personalized?	50%
10. Was the intervention modified during the treatment?	27%
11. Planned intervention adherence	9%
12. Actual intervention adherence	50%

**Table 2.** Characteristics of included studies.

Author(s), year & design	Breathlessness cause(s)	Participants	Intervention
Harrison et al. (2020)^30^ Mixed-methods (grounded theory) Uncontrolled before–after study	Chronic respiratory diseases	10 (2 male, 70 years)	Weekly dance classes, 10 weeks
Belardinelli et al. (2008)^44^ Randomised control trial	Coronary heart failure (class II & III)	130 (107 male, 59 years) Active control (*n* = 44) Passive control (*n* = 42) Dance (*n* = 44)	Dance: 3× a week, 8 weeks Active control: supervised exercise training, 3× a week, 8 weeks
Breyer et al. (2010)^45^ Randomised control trial	COPD	60 (27 male, 60.3 years) Nordic walking (*n* = 30) Passive control (*n* = 30)	Nordic walking, 1 hour, 3× a week, for 3 months
Girold et al. (2017)^46^ Randomised control trial	Acute coronary syndrome	42 (35 men, 57.2 years) Nordic walking (*n* = 21) Active control (*n* = 21)	5 45-minute sessions per week, for 4 weeks Active control group walked but without the use of poles
Kaltsatou et al. (2014)^47^ Randomised control trial	Congestive heart failure (class II & III)	51 (67.2 years) Dance (*n* = 18) Active control (*n* = 16) Passive control (*n* = 17)	8 months, 3× a week, 1-hour dance sessions Active control: aerobic & resistance exercise
Lehmann et al. (1990)^48^ Uncontrolled before–after study	Myocardial infarction	10 (10 male, 49 years)	Cross-country skiing Received instructions over 4-day period then performed a field test on the 5th day, covering approximately 7 km in 90 minutes
Nagyova et al. (2020)^49^ Pseudo-randomized, parallel-group trial	Coronary heart disease (class I to III)	86 (64 male, 59.5 years) Nordic walking (*n* = 53) Active control (*n* = 30)	3 weeks, 4 times a week Nordic walking session, 40 minutes Active control: conventional cardiovascular rehabilitation
Nohara et al. (1990)^50^ Uncontrolled before–after study	Coronary heart disease	185 (58.9 years)	3× a week. 3 sets of 20-minute sport programs are undertaken with 10-minute rest between them
Vehí et al. (2016)^51^ Uncontrolled before–after study	Coronary heart disease	23 (15 male, 69 years)	2 weekly sessions of Nordic walking led by 2 instructors for 1 hour and for 1 year
Vordos et al. (2017)^52^ Randomised control trial	Congestive heart failure (class I & II)	40 (27 male, 73.2 years) Dance (*n* = 20) Passive control (*n* = 20)	3× a week, 40–65 minutes, Greek traditional dances, intensity gradually increased
MacBean et al. (2017)^55^ Uncontrolled before–after study	COPD	5 (2 male, 61–76 years)	3 1-hour dance sessions
Anekwe et al. (2017)^56^ Randomized cross-over study	COPD	25 (13 male) COPD (*n* = 15) Healthy control (*n* = 10)	Participants performed 2 6-minute walk tests, 1 with activator poles (Nordic walking) and 1 without
Lins et al. (2003)^54^ Uncontrolled before–after study	Coronary heart disease	33 (33 male, 61 years)	Swimming. 200–250 m
Aliani et al. (2012)^53^ Randomised control trial	COPD	11 (64.2) Nordic walking (*n* = 5) Active control (*n* = 6)	Daily 30-minute session of Nordic walking for 5 days a week for a total period of 3 weeks
Sabrià et al. (2019)^57^ Uncontrolled before–after study	Idiopathic pulmonary fibrosis	21	12 weeks of Nordic walking training, 1-hour sessions
Wshah et al. (2019)^58^ Uncontrolled before–after study	COPD	20 (7 male, 73.4 years)	1-hour dance classes, twice a week for 8 weeks
Kokubo et al. (2018)^59^ Uncontrolled before–after study	Cardiovascular disease	19 (8 male, 68.3 years)	1-hour dance classes
Reed et al. (2021)^60^ Randomised control trial	Coronary heart disease	130 (110 male) Nordic walking (*n* = 43) Medium-intensity continuous training (*n* = 44) High-intensity interval training (*n* = 43)	Twice weekly for 12 weeks: 60-minute Nordic walking 60-minute medium-intensity continuous training 45-minute high-intensity interval training
Volodina et al. (2019)^61^ Randomised control trial	Acute coronary syndrome	69 (57.9 years) Nordic walking (*n* = 34) Active control (*n* = 35)	3 weekly sessions for 12 weeks, patients walked for 35–40 minutes Active control: traditional cardiac rehabilitation
Philip et al. (2021)^65^ Qualitative (thematic analysis)	Chronic respiratory diseases	19 (7 male) Patients (*n* = 11, 43 years) Staff (*n* = 8, 41 years)	20–40 minutes, dance taster sessions
Philip et al. (2020)^63^ Qualitative (thematic analysis)	Chronic respiratory diseases	8 (2 male, 75 years)	2 years of attendance, weekly dance sessions lasting 75 minutes
Maskarinec et al. (2015)^64^ Qualitative (thematic analysis)	Coronary heart disease	20 (10 male, 50–81 years)	12-week hula dance intervention, 3 classes per week

**Table 3.** Quality appraisal – Downs and Black.

	Reporting	External validity	Internal validity (bias)	Internal validity (confounding)	Power	Total
Question numbers	1–10	11–13	14–20	21–26	27	
Maximum score	11	3	7	6	1	28
Belardinelli et al. (2008)^44^	8	0	6	2	1	17
Breyer et al. (2010)^45^	7	0	5	4	0	16
Girold et al. (2017)^46^	7	0	6	3	1	17
Harrison et al. (2020)^30^	8	0	5	3	0	16
Kaltsatou et al. (2014)^47^	8	2	6	4	0	20
Lehmann et al. (1990)^48^	6	0	5	2	0	13
Nagyova et al. (2020)^49^	9	0	5	2	1	17
Nohara et al. (1990)^50^	7	2	4	2	0	15
Vehí et al. (2016)^51^	9	2	5	2	0	18
Vordos et al. (2017)^52^	7	0	5	4	1	12
Aliani et al. (2012)^53^	5	0	2	1	0	8
Lins et al. (2003)^54^	7	2	5	2	0	16
MacBean et al. (2017)^55^	5	0	3	0	0	8
Anekwe et al. (2017)^56^	7	0	3	1	0	11
Sabrià et al. (2019)^57^	4	0	5	0	0	9
Wshah et al. (2019)^58^	8	0	4	0	1	13
Kokubo et al. (2018)^59^	8	2	5	2	0	17
Reed et al. (2021)^60^	9	0	6	4	1	20
Volodina et al. (2019)^61^	7	0	5	1	0	13

**Table 4.** Quality appraisal – critical appraisals skill programme.

	Qualitative studies^30,63–65^			
Qualitative checklist*	Harrison et al. (2020)	Philip et al. (2021)	Philip et al. (2020)	Maskarinec et al. (2015)
Q1. Was there a clear statement of the aims of the research?	Yes	Yes	Yes	Yes
Q2. Is a qualitative methodology appropriate?	Yes	Yes	Yes	Yes
Q3. Was the research design appropriate to address the aims of the research?	Yes	Yes	Yes	Yes
Q4. Was the recruitment strategy appropriate to the aims of the research?	Yes	Yes	Yes	Yes
Q5. Was the data collected in a way that addressed the research issue?	Yes	Yes	Yes	Yes
Q6. Has the relationship between researcher and participants been adequately considered?	Can’t tell	Yes	Yes	Can’t tell
Q7. Have ethical issues been taken into consideration?	Yes	Yes	Yes	Can’t tell
Q8. Was the data analysis sufficiently rigorous?	Can’t tell	Yes	Yes	Yes
Q9. Is there a clear statement of findings?	Yes	Yes	Yes	Yes
Q10. Was the research valuable?	Yes	Yes	Yes	Yes

Possible answers are yes/can’t tell/no.

### Adherence and safety

Adherence for sports was reported as generally high, with all dance interventions (*n* = 4) noting over 90% attendance.^44,47,52,58^ Vehí et al. reported that 48% of participants attended at least two-thirds of the 90 Nordic walking sessions organised; however, over half acquired walking poles after the programme finished.^51^ Long-term findings from Nohara et al. implementing a mixed-sports programme found 20% of participants dropped out within 1 month.^50^ A number of participants purportedly attended consistently since the beginning of the intervention, but this figure was not specified.^50^ Nohara et al. was also the only study to offer detailed reasons for non-attendance, notably, a lack of spare time after returning to work, inability to play sport mainly due to aging, inconvenient transport, low levels of satisfaction with the programme, and choosing to self-train in similar kinds of sports.^50^

Twelve studies documented whether participants reported adverse events; eight studies reported none.^30,45,46,49,54,58,59,63^ Of the remaining four studies, adverse events disclosed included premature ventricular contractions (for 8 of 44 dancers with heart failure),^44^ arrhythmias (a ‘moderate number’ in all 10 skiers who had heart disease),^48^ a percutaneous coronary intervention (1 of 43 Nordic walkers with heart disease),^60^ and a humerus fracture 2 months post-intervention (1 Nordic walker with heart disease).^51^

### Quantitative evidence

**Figure 2.**
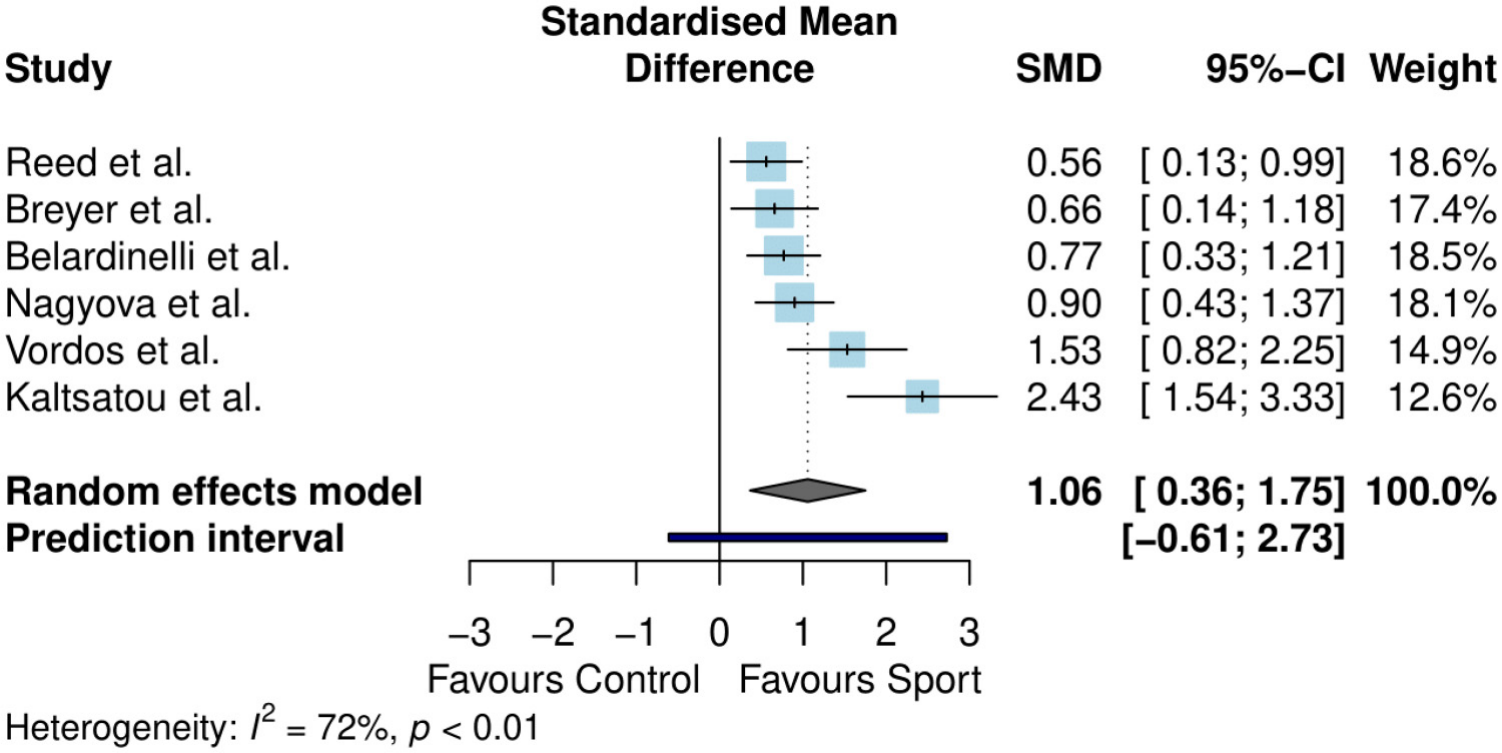
Meta-analysis results for exercise capacity.

Exercise capacity was assessed in six controlled studies (*n* = 497).^44,45,47,49,52,60^ Two measures of exercise capacity were reported: 6-minute walk distance and peak oxygen uptake. Results are outlined in Figure 2.

Exercise capacity differed significantly between sport and control conditions (standardised mean difference = 1.06, 95% confidence interval (CI) 0.36–1.75, *p* = 0.01). The between-study heterogeneity was estimated at ${\hat{\tau }}^{2}$ = 0.29 (95% CI: 0.05–2.95), with an $I^{2}$ value of 71.9% (95% CI: 35–88%). The prediction interval ranged from *g* = −0.61 to 2.73, indicating that negative intervention effects cannot be ruled out for future studies.

**Figure 3.**
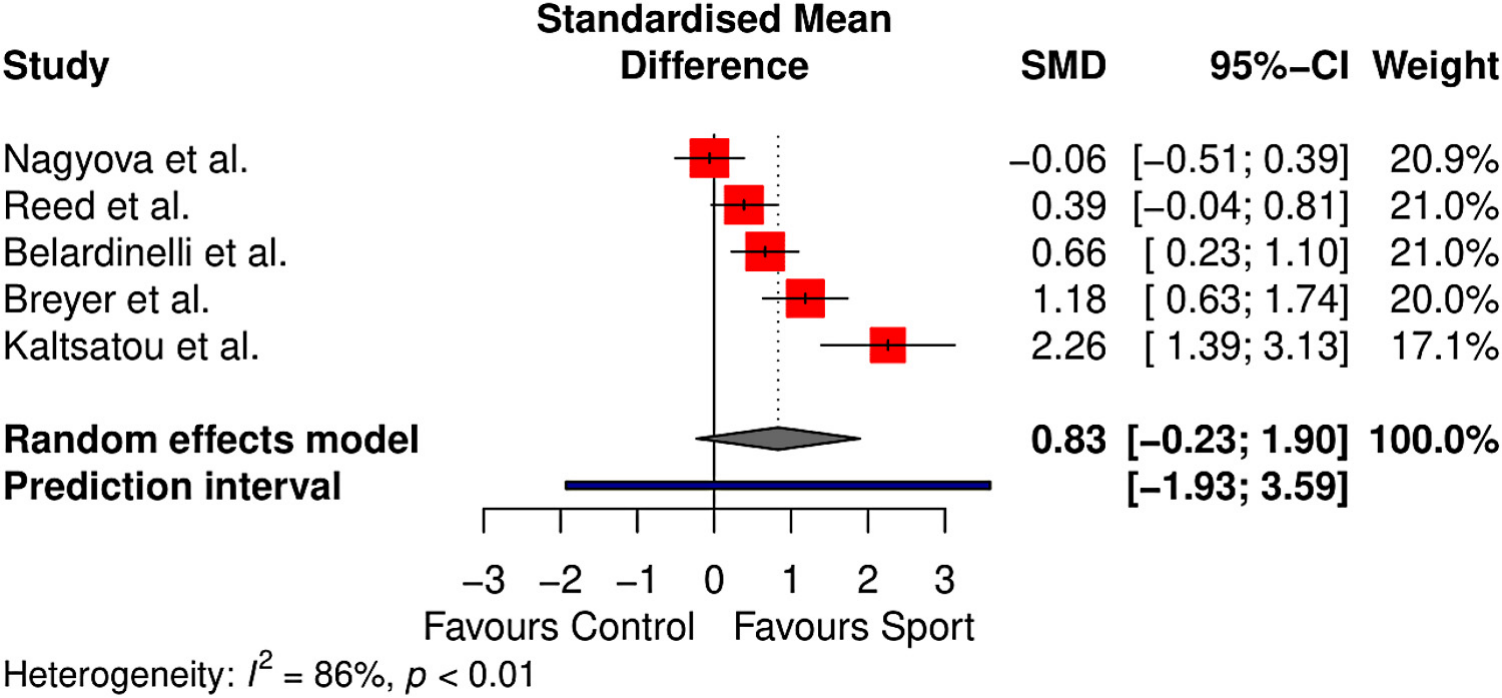
Meta-analysis results for physical health–related quality of life.

**Figure 4.**
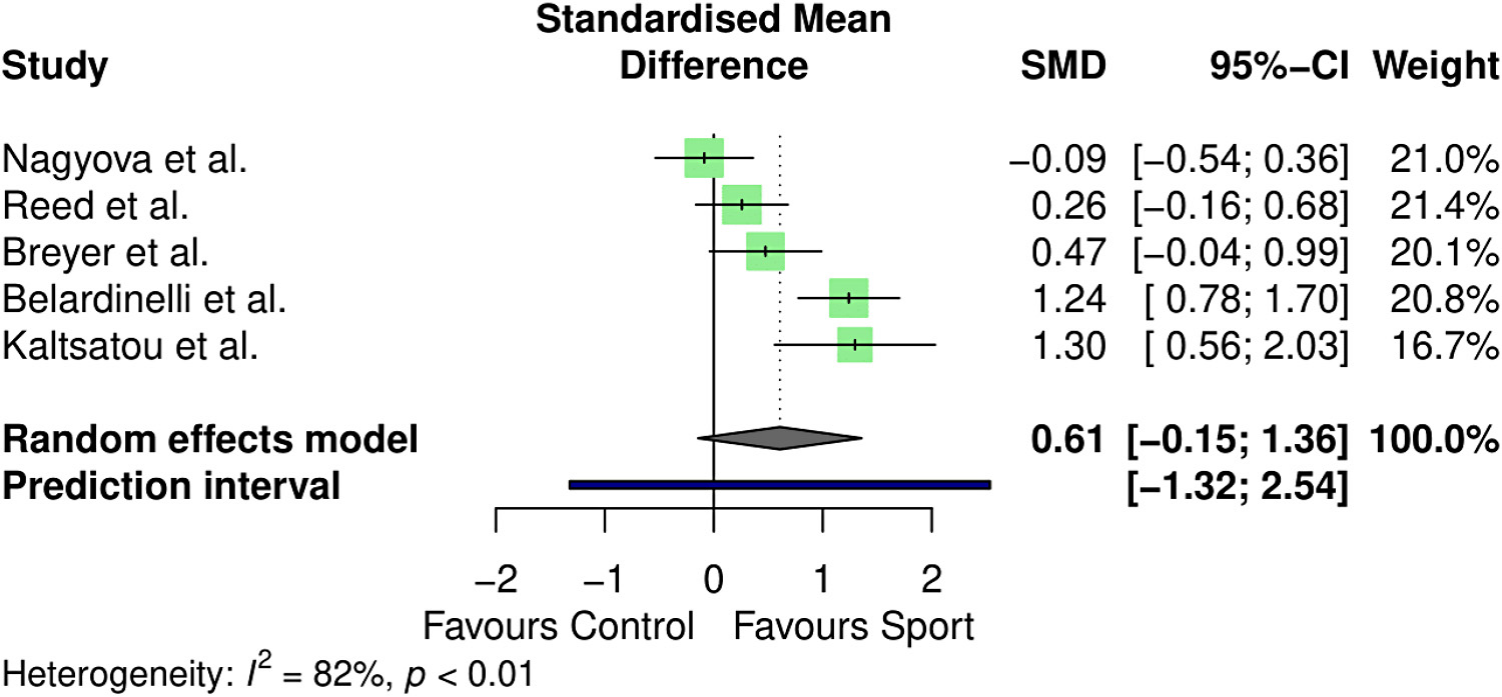
Meta-analysis results for mental health–related quality of life.

Health-related quality of life was measured in five controlled studies (*n* = 457).^44,45,47,49,60^ The 36-Item Short Form Survey and the Minnesota Living with Heart Failure Questionnaire were used to calculate health-related quality of life across studies, with separate analyses conducted for physical and mental wellbeing components. Results are outlined in Figures 3 and 4.

Physical health-related quality of life did not differ significantly between sport and control conditions (standardised mean difference = 0.83, 95% CI −0.23–1.90, *p* = 0.09). The between-study heterogeneity was estimated at ${\hat{\tau }}^{2}$ = 0.60 (95% CI: 0.16–6.41), with an $I^{2}$ value of 85.6% (95% CI: 63–94%) and a prediction interval ranging from *g* = −1.93 to 3.59.

Mental health–related quality of life likewise did not vary between conditions (standardised mean difference = 0.61, 95% CI: −0.15–1.36, *p* = 0.09). Between-study heterogeneity was estimated at ${\hat{\tau }}^{2}$ = 0.29 (0.06–3.00), with an $I^{2}$ value of 81.9% (95% CI: 58–92%) and a prediction interval of *g* = −1.32–2.54.

The remaining quantitative outcomes of interest are summarised in narrative form. Exact *p*-values are reported where available.

Exercise intensity was assessed in the majority of quantitative studies, with diverse outcome measures used. Three studies reported the *mean heart rate* (*n* = 188). Lins et al. found swimming to increase the heart rate significantly compared to heart rate at rest (Δ24 beats per minute, *p* < 0.01), and Anekwe et al. found COPD patients who walked with activator poles to have a higher mean heart rate when compared to walking without poles (*p* = 0.002).^54,56^ Belardinelli et al. did not find a significant difference in the mean heart rate when comparing dance to supervised exercise training (*p* = 0.59), although both could be deemed high intensity, given both dance and training groups reported a mean beat per minute over 110.^44^

Six studies reported the *maximum heart rate* as a measure of intensity (*n* = 342), three of which (Nordic walking and dance)^45,49,55^ report that patients achieved pre-set goals for the max heart rate, set at 70–75% of the predicted max heart rate. Belardinelli et al. compared the max heart rate in dance classes to an inactive control group and found the difference to be significant (Δ7 beats per minute, *p* < 0.05),^44^ whilst Girold et al. found no difference between Nordic walking and walking without poles after 4 weeks.^46^

Overall time spent exercising, standing, or inactive was considered in three studies (Nordic walking and dance; *n* = 151), with all finding significant results in favour of the sports interventions (*p* < 0.05).^45,47,52^ MacBean et al. (dance) was the only study to consider *perceived intensity*, discovering that participants underestimated class duration by up to 15%.^55^

Breathlessness-related outcomes were assessed across six studies (four Nordic walking,^45,53,56,57^ two dance; *n* = 132)^30,55^ using the Medical Research Council Dyspnoea Scale (*p* < 0.05),^53^ dyspnoea numeric rating scales (*p* < 0.01^45^; range of 4–9 out of 10^55^; non-significant difference to no-pole walkers),^56^ lung capacity (Δ7–14%; *p* < 0.05),^57^ and the COPD Assessment Tool (Δ−0.5).^30^

Improvements in exercise performance were evaluated across four studies (Nordic walking, skiing, and mixed-sports, *n* = 350). Outcomes encompassed blood lactate levels (*p* < 0.05),^48^ cardiovascular performance (via exercise ergometry; *d* = 0.478, *p* < 0.05),^49^ cardiac stress testing (Δ1.3, *p* < 0.001),^61^ and metabolic equivalent of task (via Bruce protocols; improvement for 58% of participants).^50^

Four lower body strength/mobility outcomes were extracted, measured across three studies (all dance; *n* = 101) via two different methods: sit to stand tests and a leg–chest dynamometer,^30,47,52^ All four outcomes changed in a positive direction (leg–chest dynamometer, Δ10.3%, *p* < 0.05^52^ and Δ81.7 pounds, *p* < 0.05^47^; sit to stand, Δ2 median reps^30^ and Δ−4.7 s, *p* < 0.05).^47^

Leg fatigue was assessed in two studies (*n* = 30) via numerical rating scales. Fatigue levels appeared to be tolerable for most with MacBean et al. (dance) reporting a fatigue range of 4–8 (out of 10, 10 being the highest fatigue)^55^ and Anekwe et al. recording similar fatigue ratings between Nordic walking and no-pole walking groups.^56^

Balance (including balance confidence) was assessed in three studies (all dance, *n* = 81), with four outcomes, including the Berg Balance Scale (Δ3, *p* < 0.05),^47^ Balance Evaluation Systems Test (*p* < 0.001),^58^ Activities-Specific Balance Confidence Scale (*p* = 0.007),^58^ and the Timed Up and Go Test (Δ0.8 seconds).^30^

Four studies calculated heart disease risk factors (dance, Nordic walking and swimming; *n* = 255) All four found risk factors to reduce significantly in favour of sports. Outcomes ranged from systolic blood pressure (Δ20 mm of mercury, *p* < 0.05),^44^ cholesterol levels (*p* < 0.05),^44^ fasting blood glucose (*p* < 0.05),^44^ mean cardiovascular risk factors (Δ−1.7, *p* < 0.0001),^51^ tissue-type plasminogen activator (Δ36 units per millilitre, *p* < 0.01),^54^ and resting heart rate (Δ−3 beats per minute, *p* = 0.041).^61^

Three studies assessed the impact of sport on depression and/or anxiety (dance and Nordic walking; *n* = 80). Results appeared equivocal, with instruments of choice encompassing the Patient Health Questionnaire-9 (Δ−6.5),^30^ Hospital Anxiety and Depression Scale (*p* < 0.01^45^; *p* > 0.05),^58^ and the Generalised Anxiety Disorder Assessment-7 (Δ0).^30^

MacBean et al. (dance) was the only study to quantitatively measure participants’ satisfaction with the intervention, using numerical rating scales (out of ten; ten being the highest satisfaction) relating to group cohesion, enjoyment, sense of achievement, and satisfaction with worth of sessions.^55^ Participants provided favourable ratings although no statistical comparisons were made. Similarly, Kaltsatou et al. (*n* = 51) was the only study to assess the impact of sport (dance) on life satisfaction (life satisfaction inventory; Δ7.8, *p* < 0.05) and intrinsic motivation (intrinsic motivation inventory; Δ0.79, *p* < 0.05) both of which improved significantly compared to baseline.^47^

### Qualitative evidence

**Figure 5.**
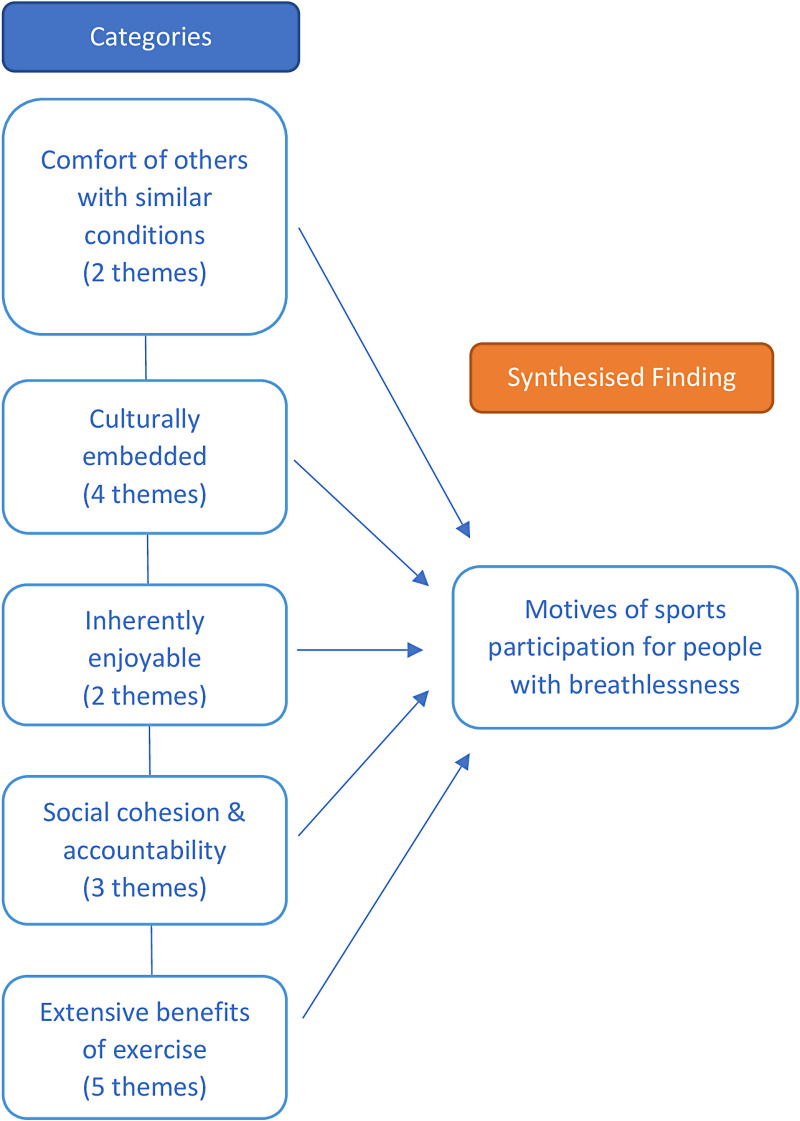
Meta-aggregation.

A visual representation of the meta-aggregation can be found in Figure 5. One synthesised finding emerged, titled *Motives of sports participation for people with breathlessness*, comprising five subordinate categories across four studies. The sport of choice in all studies was dance. No theme extracted was deemed unsupported by corresponding illustrations (see Appendix C).

[Category One] *Comfort of others with similar conditions* represented the value participants placed on exercising with others they perceived as similar to themselves with respect to symptoms and exercise ability. Participants appeared to feel reassured to be relating to those seen as comparable, with participation fostering an esprit de corps and sense of belonging. This ties in with category four, *Social cohesion*, in which the shared experience of breathlessness forged identity due to dancing together. This was juxtaposed to exercising independently in other settings such as gyms which embodied individualism; in this setting, the potential to develop camaraderie through exercise was inhibited by differing levels of ability and fitness goals.

*…the camaraderie and knowing that everybody else is in the same position. You know we all come from the same background with a cardiac problem. And so that was really a solace. (p. 112)* ^64^

[Category Two] *Culturally embedded* describes sport/dance as part of everyday life. For those with little previous experience of dance, it afforded the development of confidence, learning the steps to dances over time which they initially perceived as too complex to experience for themselves. Others described dance as the fabric of their society. It was more than a form of exercise, but rather a method of individual expression offering opportunity to educate others on matters of morality, history, philosophy, politics and local culture. Participants welcomed this knowledge acquisition stating it enhanced the dance experience, as studying dance became less about learning a number of steps but rather communicating previously underappreciated stories and practices.

*There is no culture in Uganda where there isn’t dancing. (p. 6)* ^65^

[Category Three] *Inherently enjoyable* is how participants viewed dance. Sport may appear to operate solely as a means of promoting health, but participants continued to sustain their attendance primarily because dance was a fun activity. Compared to dance, gym attendance was described as somewhat intimidating and not as engaging. Participants reported laughing throughout their dance sessions, often becoming so engrossed in the movements that they forgot they were exercising. Dancing offered a sense of achievement and contentment, perceived as time well spent.

*The dance is enjoyable that's the reason. We all laugh. It's not sort of exercise. I prefer this [dance] to exercise. There's something about the movements that seem to be more sedate and strengthening. (p. 5)* ^30^

[Category Four] *Social cohesion and accountability* refers to the social bonds formed through participation as key to the enjoyment and sustainability of dance. Many reported attending sessions frequently, not necessarily to dance, but for its social aspects. This appeared to emanate from being part of a group that cares for each other, such that when a group member struggled with breathing, peers were felt to understand this through their own lived experience, and this kindness when dancing could mitigate stress and anxieties. Evidence of care was noted when a group member was absent from a session; other dance members checked their wellbeing and encouraged their return when able.

*It's not just about dancing it's about the whole, being part of the organisation, being part of a little unit that I think I enjoy coming out. (p. 5)* ^63^

[Category Five] *Extensive benefits of exercise* were reported in all studies, citing physical and psychological improvements. Physically, participants reported feeling stronger and better able to keep up with the pace of the routines over time, noting an increased sense of balance and for some, a reduction in breathlessness symptoms, especially whilst dancing. This perceived increase in capacity led to greater confidence in their daily activities, or accepting social invitations that would have previously been declined. Through remembering the steps and moves to complex dances, alongside long spells of concentration, participants also perceived psychological improvements. In contrast, when absent from a class, participants felt their fatigue and distress more prominently.

*I just get so much more from this. I’ve noticed definition on my muscles on my arms and legs. (p. 5)* ^30^

## Discussion

This is the first systematic review examining the potential for sport in people with chronic breathlessness. Sports were well-adhered to and safe. Improvements in exercise capacity were significant; however, negative effects cannot be ruled out. There was no evidence of benefits for health-related quality of life. Other quantitative outcomes varied widely, making it difficult to draw any firm conclusions. The social/cultural aspects of sport, alongside participating with others of a similar ability, are important to ensure participation is enjoyable for people with chronic breathlessness.

Sport interventions were well-adhered to,^44,47,50–52,58^ with greater attendance levels than most cardiopulmonary programmes,^12^ likely due to the inherent enjoyability of participation. This is in contrast to cardiopulmonary rehabilitation programmes, which some perceive as boring.^66,67^ Reasons for non-adherence were only listed in one study and included a lack of spare time, inability due to aging, and choosing alternative sports^50^, reasons which somewhat differ to why patients don’t participate in rehabilitation, namely, a lack of perceived benefit/low confidence in treatment^16–18^ and accessibility.^17,19^ Adverse events were rare, minor and/or unrelated to the intervention.^30,44–46,48,49,51,54,58–60,63^

Improvements in exercise capacity are likely because participants are exercising at a moderate-to-vigorous intensity,^44,45,49,54–56,59^ similar to that observed in cardiopulmonary rehabilitation^7^ and sports with older adults.^68^ Participants also appeared able to maintain this intensity despite being self-paced, differing from cardiopulmonary rehabilitation where intensity is actively prescribed and monitored. Maintaining this intensity may be due to distraction, with focus on enjoying the sport in the moment and trying to keep up with other participants, possibly masking symptoms of breathlessness. This is supported by findings that self-reported breathlessness was either improved or maintained through participation,^30,45,53,55,56^ reductions in the unpleasantness of dyspnoea with distraction through reading,^69,70^ and similar increases in perceived self-efficacy and self-control via sports with other conditions such as Parkinson's,^71^ stroke,^72^ and multiple sclerosis.^72^

Health-related quality of life was not seen to improve, despite sports being perceived as enjoyable and socially supportive. Furthermore, previous evidence from pulmonary rehabilitation suggests health-related quality of life may be enhanced by exercising in a group.^7^ One possible explanation for this finding is a lack of power, with four of the five studies (*n* = 457)^44,45,47,49,60^ reporting increases in quality of life, yet the meta-analysis did not find a significant effect.

This review revealed domains for assessment that are rarely considered in cardiopulmonary programmes, notably, balance, co-ordination, teamwork, camaraderie and turning ability. The benefits of collaboration and collegiality through sports were prominent throughout the papers. Collective engagement was emphasised via the cultural dimensions and learning afforded through dance. Normative processes regarding peer support for exercise linked to the high levels of social cohesion and viewing participation as enjoyable rather than a chore to maintain one's health. This collective and social dimension of sport appears to contrast with exercising independently, such as walking or attending a gym. Participants directly attributed a holistic range of benefits through sport – especially psychologically and socially – however, psychosocial outcomes measured in this review were almost all secondary to the main research aims, offering equivocal results from studies with insufficient power to perform inferential tests.

In conducting this review, we note a number of limitations. As chronic breathlessness is a symptom rather than a condition in itself, the search strategy was limited to known conditions where breathlessness is likely to occur; however, this may have omitted studies including breathless participants if they were not reported overtly. Furthermore, since breathlessness as a symptom varies greatly even within conditions, levels of dyspnoea perceived by the participants in the review may not have been consistent. However, there is a growing consensus towards defining breathlessness as its own condition.^1,73^ We also acknowledge that other researchers may choose to define sport differently. Given data adequacy, meta-analyses were limited to Nordic walking and dance only. Similarly, our meta-aggregation could only utilise qualitative studies examining dance. Both constraints limit generalisability and transferability of the findings across all sports. Fewer than ten studies were included in each meta-analysis, meaning publication bias could not be assessed via funnel plots or Egger's regression test, and sub-group analysis was not conducted consonant with Cochrane's recommendations.^74^ A lack of eligible studies precluded the conduct of inferential comparisons via meta-analysis between sports.

Cardiopulmonary rehabilitation as the intervention of choice to mitigate breathlessness is typically prescribed for only 6–8 weeks and, with asynchronous start and completion dates, may not maximise common experience, a collective identity, or social cohesion to temper attrition. Where possible, physiotherapists may benefit their patients by grouping members together in rehabilitation classes to encourage social cohesion or suggest local interventions similar to the sports described here, where a level of camaraderie through shared experience can be developed more accessibly.

In conclusion, sports participation for people with chronic breathlessness was well-adhered to and safe. Exercise capacity improved, but this did not translate to improved health-related quality of life. Qualitative findings were limited to dance-based interventions, but themes emphasised the positive social cohesive elements of sports.

**Clinical messages**

- Organised sports in a social setting are well-adhered to and safe for people with chronic breathlessness.
- Meta-analyses revealed observed improvements in exercise capacity, but no similar impact on health-related quality of life domains was found.
- Participation in sports was reliably recorded at an intensity consistent with moderate-to-vigorous activity.
- Where reported, perceived breathlessness was maintained or improved by participation in sport; however, too few studies included breathlessness as a primary outcome for any confident conclusions to be drawn.
- Qualitative themes emphasised the positive, collaborative and socially cohesive elements of sports participation, offering opportunity to maintain health as enjoyable rather than to be endured.

## Appendices

**Appendix A.** Search strategy.

	#	Query
Population	S1	(MH “Respiratory Tract Diseases+”) OR (MH “Heart Failure+”) OR (MH “Myocardial Ischemia+”)
	S2	AB (Breathless* OR Shortness of Breath OR Dyspnoea OR Dyspnea OR COPD OR Chronic Obstructive Pulmonary Disease OR Asthma OR Pulmonary Rehab* OR Pulmonary Hypertension OR Pulmonary Alveolar Proteinosis OR Pulmonary Eosinophilia OR Pulmonary Fibrosis OR Respiratory Tract OR Respiratory Distress OR Respiration Disorder* OR Respiratory Insufficiency OR Respiratory Hypersensitivity OR Respiratory Abnormal* OR Respiratory Syndrome OR Lung Disease* OR Lung Injury OR Lung Neoplasm OR Bronchitis OR Emphysema OR Sarcoidosis OR Asbestosis OR Bronchiectasis OR Cystic Fibrosis OR Heart Failure OR Cardiac Failure OR Myocardial Ischemia)
	S3	TI (Breathless* OR Shortness of Breath OR Dyspnoea OR Dyspnea OR COPD OR Chronic Obstructive Pulmonary Disease OR Asthma OR Pulmonary Rehab* OR Pulmonary Hypertension OR Pulmonary Alveolar Proteinosis OR Pulmonary Eosinophilia OR Pulmonary Fibrosis OR Respiratory Tract OR Respiratory Distress OR Respiration Disorder* OR Respiratory Insufficiency OR Respiratory Hypersensitivity OR Respiratory Abnormal* OR Respiratory Syndrome OR Lung Disease* OR Lung Injury OR Lung Neoplasm OR Bronchitis OR Emphysema OR Sarcoidosis OR Asbestosis OR Bronchiectasis OR Cystic Fibrosis OR Heart Failure OR Cardiac Failure OR Myocardial Ischemia)
	S4	S1 or S2 or S3
Intervention	S5	(MH “Sports+”) OR (MH “Dancing”)
	S6	AB (Sport* OR Nordic OR Football OR Soccer OR Danc* OR Swimming OR Diving OR Surfing OR Rowing OR Polo OR Kayaking OR Canoeing OR Boating OR Surfboarding OR Ski OR Skiing OR Ski-ing OR Sailing OR Golf OR Squash OR Cycling OR Bicycling OR Biking OR Tennis OR Badminton OR Curling OR Bowls OR Hockey OR Snowshoeing OR Baseball OR Softball OR Basketball OR Boxing OR Cricket OR Gymnastics OR Martial Arts OR HapKiDo OR Judo OR Karate OR Jujitsu OR Tae Kwon Do OR Aikido OR Wushu OR Kung Fu OR Mountaineering OR Racquet* OR Lacrosse OR Racket* OR Skate OR Skating OR Volleyball OR Rugby OR Croquet OR Bowling OR Fishing OR Weightlifting OR Netball)
	S7	TI (Sport* OR Nordic OR Football OR Soccer OR Danc* OR Swimming OR Diving OR Surfing OR Rowing OR Polo OR Kayaking OR Canoeing OR Boating OR Surfboarding OR Ski OR Skiing OR Ski-ing OR Sailing OR Golf OR Squash OR Cycling OR Bicycling OR Biking OR Tennis OR Badminton OR Curling OR Bowls OR Hockey OR Snowshoeing OR Baseball OR Softball OR Basketball OR Boxing OR Cricket OR Gymnastics OR Martial Arts OR HapKiDo OR Judo OR Karate OR Jujitsu OR Tae Kwon Do OR Aikido OR Wushu OR Kung Fu OR Mountaineering OR Racquet* OR Lacrosse OR Racket* OR Skate OR Skating OR Volleyball OR Rugby OR Croquet OR Bowling OR Fishing OR Weightlifting OR Netball)
	S8	S5 or S6 or S7
Outcomes	S9	(MH “Health”) OR (MH “Program Evaluation”) OR (MH “Benchmarking”) OR (MH “Treatment Adherence and Compliance”) OR (MH “Accidental Injuries”) OR (MH “Affect”) OR (MH “Metabolic Equivalent”) OR (MH “Physical Exertion”) OR (MH “Perception”) OR (MH “Attitude”) OR (MH “Emotions”) OR (MH “Motivation”) OR (MH “Diagnostic Self Evaluation) OR (MH “Personal Satisfaction”) OR (MH “Feasibility Studies”) OR (MH “Sleep”) OR (MH “Body Mass Index”) OR (MH “Body Weight”) OR (MH “Exercise Tolerance”) OR (MH “Fatigue”) OR (MH “Posture”) OR (MH “Postural Balance”) OR (MH “Blood Glucose”) OR (MH “Cognition”) OR (MH “Memory”) OR (MH “Smoking”) OR (MH “Diet”) OR (MH “Sedentary Behaviour”) OR (MH “Alcohol Drinking”) OR (MH “Heart Rate”) OR (MH “Waist Circumference”) OR (MH “Body Fat Distribution”) OR (MH “Cardiorespiratory Fitness”) OR (MH “Blood Pressure”) OR (MH “Muscle Strength”) OR (MH “Cholesterol”) OR (MH “Mental Health”) OR (MH “Anxiety”) OR (MH “Fear”) OR (MH “Depression”) OR (MH “Social Support”) OR (MH “Loneliness”) OR (MH “Quality of Life”)
	S10	AB (Physiological OR Health OR Intensity OR Duration OR Sustainability OR Adherence OR Injur* OR Accident OR Enjoy* OR Affect OR Metabolic OR MET OR Exertion OR RPE OR Borg OR Step* OR Distance OR Cadence OR Experience* OR Views OR Meaning OR Attitudes OR Opinions OR Feelings OR Motivat* OR Triggers OR Impact OR Perceptions OR Evaluation OR Satisfaction OR Engagement OR Acceptability OR Facilitators OR Feasib* OR Sleep OR Mass OR Weight OR BMI OR Capacity OR Fatigue OR Stability OR Oxygen OR VO2 OR Exhaustion OR Blood Lactate OR Postur* OR Balance OR Sugar OR Glucose OR Cognitive or Cognition OR Smoking OR Diet OR Nutrition OR Drinking OR Alcohol OR Heart Rate OR HR OR Waist OR Circumference OR Blood Lipid OR Cardiorespiratory OR CRF OR Blood Pressure OR Strength OR Cholesterol OR Psychological OR Mental OR Social OR HADS OR Anxiety OR Stress OR Worry OR Fear OR Depres* OR Support OR Lonely OR Loneliness OR Quality OR Wellbeing)
	S11	TI (Physiological OR Health OR Intensity OR Duration OR Sustainability OR Adherence OR Injur* OR Accident OR Enjoy* OR Fun OR Affect OR Metabolic OR MET OR Exertion OR RPE OR Borg OR Step* OR Distance OR Cadence OR Experience* OR Views OR Meaning OR Attitudes OR Opinions OR Feelings OR Motivat* OR Triggers OR Impact OR Perceptions OR Evaluation OR Satisfaction OR Engagement OR Acceptability OR Facilitators OR Feasib* OR Sleep OR Mass OR Weight OR BMI OR Capacity OR Fatigue OR Stability OR Oxygen OR VO2 OR Exhaustion OR Blood Lactate OR Postur* OR Balance OR Sugar OR Glucose OR Cognitive or Cognition OR Memory OR Smoking OR Diet OR Nutrition OR Drinking OR Alcohol OR Heart Rate OR HR OR Waist OR Circumference OR Blood Lipid OR Cardiorespiratory OR CRF OR Blood Pressure OR Strength OR Cholesterol OR Psychological OR Mental OR Social OR HADS OR Anxiety OR Stress OR Worry OR Fear OR Depres* OR Support OR Lonely OR Loneliness OR Quality OR Wellbeing)
	S12	S9 OR S10 OR S11
Study Design	S13	TI (Intervention* OR Programme* OR Program* OR Project* OR Training) NOT (Systematic Review OR Meta-Analysis OR Scoping Review OR Integrative Review)
	S14	AB (Intervention* OR Programme* OR Program* OR Project* OR Training) NOT (Systematic Review OR Meta-Analysis OR Scoping Review OR Integrative Review)
	S15	S13 OR S14
	S16	S4 AND S8 AND S12 AND S15

**Appendix B.** Summarised study findings.

Appendix B	
Summarised study findings	
Author(s) & year	Findings
Harrison et al. (2020)	5 themes identified: (1) Bonding among the group (2) Sense of learning and being together (3) Fun / feeling they were not ‘exercising’ (4) The impact of dance itself (5) Commitment among participants to staying well No inferential statistics, pre;post (median (IQR)): *6MWT (min):* 280.5 (254.5–312.5); 291.0 (263.5–321.0) | *TUG (s):* 10.8 (8.7–12.7); 10.0 (7.0–11.0) *30-STS:* 10.0 (9.0–12.3); 13.0 (11.0–14.5) | *CAT:* 27.5 (23.0–32.0); 27.0 (25.0–28.5) *PHQ-9:* 14.5 (5.0–17.0); 8.0 (4.5–10.5) | *GAD-7:* 5.0 (1.8–12.0); 5.0 (0.5–6.0)
Belardinelli et al. (2008)	Premature ventricular contractions in 8 patients during dancing, 6 for exercise training. % of *sessions attended* (Dance; exercise training): 91.8%; 77.8%, *p* = .02 *Mean HR, bpm* (SD): Active Control, 111 (15); Dance, 113 (19) *p* = .59 **p* < .05 vs. negative control *Peak HR, bpm* (SD) [8 weeks]: Active Control, 135 (16)* | Dance, 138 (16)* |Negative control, 131 (14) *Peak V02, ml/(kg-min)* (SD) [8 weeks]: Active Control, 19.6 (4.5)* | Dance, 19.5 (5.0)* | Negative control, 15.8 (4.5) *Systolic blood pressure, mm Hg* (SD) [8 weeks]: Active Control, 168 (16)* | Dance, 165 (20)* | Negative control, 150 (20) *High-density lipoprotein cholesterol, mg/dL* (SD): Active Control, 33 (10); 37 (11)* | Dance, 35 (9); 39.5 (8)* *Fasting Blood Glucose, g/dL* (SD): Active Control, 101 (13); 91 (12)* | Dance, 98 (12); 88 (11)* *MLHFQ:* Active Control: 53;40* | Dance: 55;39* | Negative Control: 58;57
Breyer et al. (2010)	All patients achieved the pre-set goal *for maximum HR* to ensure training efficiency (> 75% of the initial maximum HR), even though walking at different speeds according to the severity of COPD No (serious) adverse events reported *Movement intensity, m/s2* (SD) [3-months] increased compared to baseline (Δ: +0.40 (0.14), *p* < 0.01) and control (*p* < 0.01) Nordic Walking group increased *walking time* (Δ: +14.9 (1.9) min/day, *p* < 0.01) and *standing time* (Δ: +129 (26) min/day, *p* < 0.01), at the expense of *sitting time* (Δ: -128 (15) min/day, *p* < 0.01) At nine months, *walking time* remained increased (Δ: +9.2 (2.9) min/day, *p* = 0.036), and *sitting time* decreased (Δ: -101 (36) min/day, *p* = 0.032) compared to baseline and controls (*p* < 0.01) *6MWD* (SD), increased in the Nordic walking group compared to baseline (Δ: +79 (28) metres; *p* < 0.01) and control (*p* < 0.01) *Borg dyspnoea score* improved in the Nordic walking group compared to baseline (*p* < .01) and remained decreased after six and nine months (*p* < 0.01). Scores remained unchanged for the control group *HADS* in the Nordic Walking group decreased compared to baseline and to controls (*p* < .01) The *SF-36* physical component summary score increased in the Nordic Walking group compared to baseline and control (*p* < 0.01). The mental component summary remained unchanged in both groups
Summarised study findings (Continued)	
Author(s) & year	Findings
Girold et al. (2017)	No significant adverse effects No difference between groups in maximum heart rate after 4 weeks Both groups showed improvement in in *6MWT* (SD): 65 (29) m (*p* < 0.001) and 25 (35) m (*p* < 0.05) Nordic walking group covered greater distance during the *6MWT* (40 (21) m) than the walking group (*P* < 0.001)
Kaltsatou et al. (2014)	The dance group attended 96.3% of the offered exercise sessions and had the lowest drop-out rate (5.3%) No *adverse effects* reported **p* < .05 baseline vs follow-up & vs negative control | Baseline; Follow-up *Sit to Stand test (s)* (SD): 18.0 (1.8); 14.3 (1.1)* *Berg balance scale* (SD): 52.8 (1.9); 55.8 (0.4)* *Isokinetic strength testing (lb)* (SD): 209.7 (29.9); 291.4 (27.9)* *Exercise time (min)* (SD): 7.0 (0.8); 10.4 (1.2)* *VO2Peak (ml/kg/min)* (SD): 19.5 (1.7) 26.1 (2.6)* *SF-36* (SD): 82.1 (2.8); 88.6 (2.4)* *Life Satisfaction Inventory* (SD): 42.8 (5.5); 50.6 (5.2)* *Intrinsic Motivation Inventory* (SD): 3.08 (0.1) 3.87 (0.5)*
Lehmann et al. (1990)	Moderate number of *Arrhythmias* observed *Blood lactate* levels after 45 (3.7 (2.2) mmol/L) and 90 mins (2.9 (1.1) mmol/L) increased significantly (*p* < 0.05)
Nagyova et al. (2020)	All patients achieved the pre-set goal for *maximum HR* to ensure training efficiency (>75% of the initial maximum HR), even though walking at different speed levels according to the severity of CAD The rating of *perceived exertion* in participants was within the mild/moderate range (12 to 14 points) on the 0-20 Borg scale No *adverse events* reported The difference in change between Nordic walking and the control group in *cardiovascular performance* (assessed by exercise ergometry) was significant *P* < 0.05 with a small effect size (Cohen's d = .478) *Tolerance of physical activity*, expressed in achieved METs, was significantly better in the NW group compared to controls: (ΔMETs: +9.8% vs. +1.5%,, *P* < 0.01, medium ES, d = 0.565) 6MWT (Nordic walking vs. control): (Δ: +8.3% vs. +5.1%, *P* < 0.05, small ES, d = 0.468) No differences found for the *SF-36*
Nohara et al. (1990)	21.8% of patients dropped out within 1-month | Some attended for 8+ years since the beginning of the program Common reasons for non-attendance: (1) Lack of spare time after returning to work (2) Inability to play sports mainly due to old age. (3) Inconvenient transport to attend 3x a week. (4) Erroneous satisfaction contributing to voluntary withdrawal (5) self-training with similar kinds of sports Exercise tolerance via a Bruce protocol improved in 58% of participants, 36% showed no change.
Summarised study findings (Continued)	
Author(s) & year	Findings
Vehí et al. (2016)	One patient suffered a humerus fracture, most however assimilated with the technique early on 48% attended two-thirds of the 90 sessions, more than half acquired poles after the programme ended *Mean difference of CVRFs*: − 1.70 (SD 1.52, 95% CI − 1.04 to − 2.35; *p* < .0001) Significant decrease in REGICOR risk of 3 points in the group of patients with MS EQ-5D showed an overall improvement trend in patients attending more than 50% of sessions
Vordos et al. (2017)	Dance attendance was 91.25%. The dance showed significant improvements in: *walking distance* (10.0%; *p* < .05), *lower limb strength* (10.3%; *p* < .05), *plyometric jump; height* (13.9%; *p* < .05), *countermovement jump; height* (10.7%; *p* < 0.05) and *squat jump height* (10.5%; *p* < .05) *Strength of lower limbs* in the dance group was correlated with *plyometric jump* (r = 0.79, *p* < .01), *countermovement jump* (r = 0.83; *p* < 0.01) and *squat jump* (r = 0.86; *p* < .01)
Aliani et al. (2012)	Nordic walking group had significant improvements in the 6MWT and MRC [pre-post] (*p* < .05), whereas the active control only had an improvement in the MRC (*p* < .05) Improvement in the SGRQ (*p* < .05)
Lins et al. (2003)	All participants had good tolerance for swimming. There were no angina attacks in any of the patients. *Heart rate* increased significantly during swimming (*P* < .01), from 68 (pre) to 92 bpm (post). Tissue-type *plasminogen activator* (0.14 (0.03) vs. 0.50 (0.06) U/ml, *p* < .01) *Thromboplastin time* (12.8 (0.1) vs. 12.1 (0.1) s, *p* < .01)
MacBean et al. (2017)	*Peak HR* reached 79-95% of predicted maximum, and was maintained above 70%max for median 26% of class duration Individual participants’ peak NRS values for dyspnoea ranged from 4–9, and for leg fatigue from 4–8 (out of 10) participants underestimated class duration by up to 15% All participants showed improved mood (from median (range) 5 (4-7) to 7 (5-7)), sense of group cohesion (6 (3-6) to 7 (5-7)), deemed the sessions worthwhile (7 (6-7)), and expressed enjoyment (7 (6-7)) and a sense of achievement (6 (3-7)) on completion
Anekwe et al. (2017)	6MWT distance was shorter with AP (468.7 ± 133.7 m) compared to NP (429.7 ± 147.4 m, *P* = .012) in COPD; no difference emerged in the control group (572.9 ± 55.5 and 539.7 ± 64.6, respectively) Dyspnoea and leg fatigue ratings were similar for NP and AP in the two groups In individuals with COPD, walking with AP resulted in a higher oxygen consumption (*P* < .001), energy expenditure (*P* < 0.001), heart rate (*P* = .002), minute ventilation (*P* = .001) and respiratory rate (*P* < .001)
Sabrià et al. (2019)	An increase of ≥25 metres in 6MWT at 12 weeks of training was observed in 43% of patients. Despite pulmonary function test decline in 4 patients, 3 of them increased metres walked without increasing. Total Lung Capacity improved between 7-14% in 6 patients (*p* > 0.05).
Wshah et al. (2019)	95% completion rate, 1 participant withdrawing because of an unrelated illness, mean attendance rate was 78%, no adverse events associated with the dance program Fun, enjoyment, and social aspects perceived as the best features of the classes by participants Significant improvements in the 6MWT (*P* = .034; ES = 0.51), BESTest (*P* < .001; ES = 1.29), and ABC scale (*P* = .007; ES = .68) after 8-week intervention No changes in HADS anxiety or depression scores
Kokubo et al. (2018)	Mean MaxHR was 136.5 (18.3) bpm, median rating of perceived exertion (IQR) reported after the dance exercise trials was 11 (9–13), no signs of overexertion during the dance trial
Reed et al. (2021)	Adverse Events: 1 percutaneous coronary intervention for NW Greater improvement in 6MWT distance for patients engaging in NW than MICT (F = 4.962, *p* = .029) and HIIT (F = 4.039, *p* = 0.048) Significant main effects of time showed increases in HeartQoL (F = 12.908, *p* < 0.001)
Volodina et al. (2019)	Decrease in resting heart rate (from 74.5 (11.2) to (71.5 (11.0; *p* = .041) Increase in MET (from 4.0 ± 1.0 to 5.3 ± 2.0; *p* < .001) Cardiac stress test total test time (from 4.0 ± 1.0 to 5.3 ± 2.0; *p* < .001)
Philip et al. (2021)	4 themes identified: Music and dance as (1) central components of daily life; having an (2) established role supporting health and wellbeing; and perceived as having (3) strong therapeutic potential in respiratory conditions. However, the potential realisation of this ‘strong potential’ was dependent upon theme (4) modulating demographic considerations of cultural and religion, and age.
Philip et al. (2020)	4 themes identified: Dance as (1) a holistically beneficial activity, with physical and psychosocial health benefits including improved or maintained physical fitness and psychological well-being, and reduced need for healthcare; (2) an integral part of their life; (3) an enjoyable activity; and (4) a source of deep social cohesion.
Maskarinec et al. (2015)	Patients’ enthusiasm for the therapeutic modality was overwhelmingly positive. All 20 participants narrated thoughtful, detailed reflections on the effects they perceived of the intervention on their physical, mental, spiritual, and social well-being. Many of those interviewed noted that it felt therapeutic to meet other people who were also attempting to recover from a similar health event and to participate with them in a common activity. Participants emphasised that the cultural aspects of the intervention were very important to them.

6MWT, six-minute walk test; TUG, timed up and go; 30-STS, 30 s sit to stand test; CAT, COPD assessment tool; PHQ-9, Patient Health Questionnaire; GAD-7, Generalised Anxiety Disorder Assessment; MLHFQ, Minnesota Living with Heart Failure Questionnaire; COPD, Chronic Obstructive Pulmonary Disease; 6MWD; six-minute walking distance; HADS, Hospital Anxiety and Depression Scale; SF-36, 36-Item Short Form Survey; VO2 peak, peak oxygen consumption; METs, Metabolic equivalents of task; CVRFs, Cardiovascular risk factors; REGICOR, Registre Gironí del Cor (Spanish cardiovascular disease risk scale); EQ-5D, Health-related quality of life measure; MRC, Medical Research Council Dyspnoea Scale; SGRQ, St. George Respiratory Questionnaire; NRS, Numerical Rating Scale; BESTest, Balance Evaluation Systems Test; ABC, Activities-specific balance confidence scale; HeartQoL, Heart related quality of life questionnaire

**Appendix C.** Themes and illustrations.

Theme / key finding	Example illustrations from text
Bonding among the group (Harrison et al., 2020) Credible	“It's the social side of it. I like to do the dances, everybody is nice, everybody knows everybody.”
Sense of learning and being together (Harrison et al., 2020) Unequivocal	“Today's class was good, we all got up and we all participated and I think we all did well. We laugh, even though I am terribly out of breath today, still enjoyed it, and we’ve laughed at each other and laughed at ourselves.”
Fun / feeling they were not ‘exercising’ (Harrison et al., 2020) Unequivocal	“The dance is enjoyable that's the reason. We all laugh. It's not sort of exercise. I prefer this [dance] to exercise. There's something about the movements that seem to be more sedate and strengthening.”
The impact of dance itself (Harrison et al., 2020) Unequivocal	“When I’m dancing, I’m no longer myself. I’m my right and my left, I’m my movement…I forget I’m ill.” “We can concentrate on our dancing and not on our breathing.” “My balance wasn’t very good to start with and I feel not that it got much better.” “I just get so much more from this. I’ve noticed definition on my muscles on my arms and legs.” “Each week I’m looking forward to dancing on Friday.”
Commitment among participants to staying well (Harrison et al., 2020) Unequivocal	“I feel like a kid in the playground again.” “It's playful, it just relaxes you.”
Music and dance as central components of daily life (Philip et al., 2021) Unequivocal	“Music really is everywhere for us … Music is really part of our fabric as a society….when they play a song everyone identifies to and everyone is getting up and just dancing …” “Music is very important in our society because it gives messages, it educates through music you are able to know what is good, what is bad, what can be done, what happened in the past, what will happen in the future, all can be delivered through music.”
Music and dance having an established role supporting health and wellbeing (Philip et al., 2021) Unequivocal	“Instead of getting angry, [I] would try and find comfort in singing and dancing to control [my] anger.” “Now [dance] has been taken up as one of the things that's used for physical exercises.” “You are joining other people. You know, when you are a people orientated person, when you find people that are happy, you also become happy.”
Music and dance perceived as having strong therapeutic potential in respiratory conditions (Philip et al., 2021) Unequivocal	“… I think if someone who is told that if you dance, if you sing is going to improve your health I believe they will have no problem taking part of it.” “No amount of medicine can give you that human connection, which is a very important part of management.” “You go beyond your tidal volume, in terms of reaching out your respiratory effort … if they keep doing this song then every other time they have some incremental effort required of their respiratory muscles.” “I was feeling a bit happier because I feel like I could breathe a bit better.”
Modulating demographic considerations of cultural and religion, and age (Philip et al., 2021) Unequivocal	“Dancing has no formula, it has no pattern. It's not a matter of, oh you must conform. Each one has their own dance. I believe that if I was dancing with you, you have your own style of dancing, and I have my own style of dancing.” “There is no culture in Uganda where there isn’t dancing.” “The old people they still love their music. Where it's a story telling song or it's something to harmonise and move or advance excitement at a party. Yeah generally the young people of course they love it. Dancing and shaking around.” “For those who are a bit weak, to know they can rest, when the body feels that it is tired. I thought that was good.”
Dance as a holistically beneficial activity (Philip et al., 2020) Unequivocal	“It's a bit of everything, afternoon social activity, because everyone has tea when they finish, or pub. It's a bit of exercise, a bit of, entertainment, it's a bit of, overall, learning.” “Also when the music comes on, you, about three beats and you’re like, ‘oh’, so I like that because you’re like, it's about maintaining that brain, that grey brain matter, yeah. And basically keeping you active.” “You feel you can do more things. So, if there's an offer coming up for an outing to a garden all day, I know that I’m going to go.” “Psychologically the illness has not taken over… because you’re occupying your mind on something completely different, so you can’t say ‘oh I can’t do that, I can’t breathe’, I can have a go. That's what life's all about at the end of the day.” “I haven’t had a bad bronchitis episode since I’ve been doing it.”
An integral part of their life (Philip et al., 2020) Unequivocal	“It's just part of my life now” “I was your typical wallflower …. I never really got into dancing at all. Till now.” “I’m learning something new and it's also, you come in and learn a dance that six weeks ago you never thought you’d get your feet around.”
An enjoyable activity (Philip et al., 2020) Unequivocal	“There is this immense feeling of contentment, and it's great. You know, when you’ve made an effort, you’ve done it, and jolly good. And that's it.” You can feel it. You get the buzz. You have a good laugh and, apart from doing your dancing you’re having fun, even with the dancing you’re having fun.” “I won’t go to the gym where there's a lot of younger people than myself. Because they can be intimidating. So, I won’t do nothing like that.”
Source of deep social cohesion (Philip et al., 2020) Unequivocal	“It's not just about dancing it's about the whole, being part of the organisation, being part of a little unit that I think I enjoy coming out.” “As a group, you know, we obviously bonded with each other, well we know each other, it is, you know it's something that…. I think we all enjoy it.” “We kind of understand each other when we can’t breathe.” “But I think that for me it's about forgetting what's wrong with me until I actually go home.” “If you go to the gym, you’re just doing your own thing, whereas here we are supporting each other.”
Physical, mental, spiritual, and social well-being (Maskarinec et al., 2015) Unequivocal	“Hula represents, I think more than just a physical entity of learning the steps and the movements with the rest of your body, with the hands and everything. It's a mental thing and a spiritual thing.” “It got you working mentally: remembering the steps, and remembering the moves, and concentrating on the beat, and everything. . . . All of us just got stronger as the class went on. When you’re able to lift your hands higher, when you’re able to just keep up with the pace, you know that you’re getting stronger, physically more fit.” “If I don’t do the hula, then I’m so lazy and have no motivation. After the class I feel I can take on the world.”
Therapeutic to meet similar people (Maskarinec et al., 2015) Unequivocal	“the camaraderie and knowing that everybody else is in the same position. You know we all come from the same background with a cardiac problem. And so that was really a solace.” “I thought I was the sickly one. I was—but when, when I got to meet everybody got to know all their . . . . it was like—I was there with a toothache.” “The first thing, when we see each other, we say ‘eh, we’re still alive!’ with a big smile. That says it all.”
Cultural aspects viewed as very important (Maskarinec et al., 2015) Unequivocal	“You gain a much greater appreciation for the Hawaiian culture. . . . I think there's an invisible part of it that you don’t really see.” “Another thing too is that the teacher explained each session of what it meant, the rain, the flowers, how she felt, why she wrote the song that way so when you started dancing you could really express it in your movement and in your hands.”
